# Indigenous neonatal feeding and bathing practices of caregivers in Vhembe District, Limpopo province

**DOI:** 10.4102/hsag.v26i0.1632

**Published:** 2021-11-29

**Authors:** Patience M. Tulelo, Fhumulani M. Mulaudzi

**Affiliations:** 1 Department of Nursing Science, Faculty of Health Sciences, University of Pretoria, Pretoria, South Africa; 2 Faculty of Health Sciences, University of Pretoria, Pretoria, South Africa

**Keywords:** caregiver, indigenous neonatal care practices, neonate, indigenous knowledge holder, traditional health practitioners

## Abstract

**Bac kground:**

Caregivers are offered health information on neonatal care before they are discharged from the healthcare facilities after giving birth. However, they continue to feed and bath neonates in ways that are informed by indigenous traditions. Notably, these ways include the provision of supplementary feeds before 6 months and bathing the neonate as early as possible, which are practices that contradict the World Health Organization (WHO) recommendations of neonatal care.

**Objectives:**

This study aimed to explore and describe the indigenous neonatal feeding and bathing practices of caregivers in Vhembe District, Limpopo province.

**Setting:**

This study was conducted in Limpopo province at Vhembe District, Makhado Municipality.

**Methodology:**

A qualitative, explorative and descriptive enquiry was used to conduct 18 semi-structured individual interviews to explore and describe their indigenous neonatal feeding and bathing practices. Purposive and snowball sampling methods were used to select participants. Creswell’s method of data analysis was used to analyse data. Ethical principles were maintained.

**Results:**

Two themes with sub-themes resulted from data analysis presenting indigenous neonatal feeding practices and indigenous neonatal bathing practices.

**Conclusion:**

This study revealed that caregivers use indigenous neonatal feeding and bathing practices across age groups and social standing. Younger mothers receive guidance from older women in their families or community. Midwives should know the indigenous neonatal feeding and bathing practices of the communities they serve to offer relevant culture-sensitive health education.

**Contributions:**

This study contributes to the creation of knowledge about indigenous neonatal care practices amongst mothers and caregivers.

## Introduction and background

Health outcomes of neonates are not only determined by their biological factors but also by the socio-cultural environment of their place of birth (Latha, Kamala & Srikanth 2017:869). Caregivers’ beliefs around pregnancy and post-natal care are shaped by their indigenous cultural origins (M’soka, Mabuza & Pretorius 2015:4; Ngunyulu, Mulaudzi & Peu 2015:2). People of the Vhembe District are not an exception as their neonatal feeding and bathing practices are informed by their indigenous traditions. Early neonatal feeding and bathing practices are considered important for cultural purposes such as the need to remove the vernix as soon as possible since it is considered dirty (Bee, Shiroor & Hill 2018:12; Memon et al. 2019:329). As much as it is important to bath the neonate after birth according to culture, thermal care and delayed neonatal bathing are recommended to prevent complications of hypothermia (World Health Organization [WHO] 2013:25).

Several studies have been conducted on indigenous neonatal feeding and bathing practices in other countries. These studies highlight that neonates ought to be provided supplementary feeds before the recommended age of 6 months according to traditional practices (Aborigo et al. 2012:8; Asim et al. 2014:665). Furthermore, neonatal feeding practices have the potential of protecting the neonate from illnesses or introducing illnesses to the neonate depending on the quality and type of feeds (Brahm & Valdes 2017:15).

Studies have highlighted that in most rural traditional contexts, neonates are given supplementary feeds and are bathed soon after birth despite being aware of the importance of exclusive breastfeeding and delayed bathing (Asim et al. 2020:8; Sacks et al. 2015:6). In South Africa, the Department of Health has put in place measures to educate caregivers on exclusive breastfeeding to promote neonatal health (Department of Health 2014:1). Despite these initiatives, caregivers continue to feed and bathe the neonate earlier than recommended as soon as they are discharged from the health institutions. The practices of feeding and bathing the neonates are mostly influenced by older family members after childbirth (Sutan & Berkat 2014:2).

### Problem statement

Early neonatal feeding and immediate neonatal bathing are practices that can pose a risk to the health of the neonate and are not recommended by the WHO (2013:25), yet they are common in Vhembe District. According to the researcher’s best knowledge, there is limited data concerning indigenous neonatal feeding and bathing practices in South Africa. It was against such a background that the researcher intended to explore and describe the indigenous neonatal feeding and bathing practices of caregivers through a qualitative study.

### Research purpose

This study explored and described the indigenous neonatal feeding and bathing practices of caregivers in selected villages of Makhado Municipality in Vhembe District, Limpopo province.

### Research question

The question that guided the study was ‘what are the indigenous neonatal feeding and bathing practices of mothers and caregivers in Vhembe District, Limpopo province?’

## Research methods

### Design and setting

A qualitative, exploratory, descriptive and contextual design was used to explore and describe the indigenous neonatal feeding and bathing practices of caregivers in Makhado Municipality, Vhembe District of Limpopo province. Participants were recruited at a specific hospital and clinic. Home interviews were conducted in selected villages around the Makhado Municipality. Semi-structured individual interviews were used to collect data.

### Population and sampling

The study comprised mothers and caregivers. Indigenous knowledge holders (IKHs) and a traditional healthcare practitioner who specialise in neonatal care were also included in the study for verification of data. Purposive and snowball sampling techniques were used to select participants. The number of participants who met the sampling criteria was initially insufficient, necessitating the researcher to seek more participants. In this case, the researcher used the snowball sampling technique. The researcher located and recruited caregivers, IKHs and the traditional health practitioners (THPs) through some of the mothers during home visits. Participants were asked whether they knew of a household with a neonate and of any woman who was caring for a neonate. Many of them eagerly provided the needed information, which enabled the researcher’s eventual identification and selection of the caregivers, IKHs and THPs. One of the IKHs was an aunt to the mother and she was also assisting with the care of the neonate. The other IKH and the THP were not related to any mother but were known to some mothers and caregivers as they rely on them for advice on indigenous neonatal care practices.

For eligibility to take part in the study, participants had to be residences of Makhado Municipality, be 20 years of age and above and having given birth or taken care of a neonate aged 3 days to 1 month. The sample size was controlled by data saturation, which was reached at Participant 13. However, the researcher continued to the next five participants for purposes of generating new data, which did not materialise as anticipated. In all, 18 participants were interviewed of whom 9 were mothers, 6 were caregivers, 2 were IKHs who specialise in the prevention and treatment of childhood illnesses and one was a THP. Table 1 indicates the demographics of the participants.

**TABLE 1:** Demographic information of the sample (*n* = 18).

Participant	Age	Parity	Educational level
P1CM	36	5	Grade 12
P2CM	25	2	Grade 12
P3CM	26	2	Grade 12
P4CM	35	4	Grade 12
P5HM	29	2	†N6
P6HM	39	3	Grade 12
P7HM	22	2	Grade 12
P8HM	40	5	Grade 12
P9HC	59	3	Grade 4
P10HM	40	5	†College certificate
P11HC	49	4	Grade 12
P12HC	63	8	Grade 1
P13HC	76	5	Grade 2
P14HC	45	2	†College certificate
P15HC	56	4	Grade 8
P16HIKH	77	4	No schooling
P17HIKH	72	7	No schooling
P18HTHP	37	2	†College certificate

† , Education after completion of school.

### Recruitment of participants

Participants were recruited from two locations: a post-natal clinic at a specific hospital and a specific clinic. The researcher requested a meeting with the managers after gaining permission from the facilities to provide information about the planned study. The operational and facility managers of the chosen facilities provided a schedule of days on which the well-baby and post-natal services were offered. This enabled the researcher to schedule visits to the institutions to recruit participants. The participants were approached on their follow-up visit days for recruitment by the researcher. Those who were willing to be interviewed in the institution signed the informed consent form after the information session and were interviewed on the same day. Those who agreed to be interviewed at their homes were followed up at their homes at a time convenient to them. Some of the caregivers were recruited at facilities whilst accompanying mothers for follow-up visits and others were recruited at the mothers’ homes during home interviews. The IKHs and THP were recruited at their residences. They were given a briefing about the study and they agreed to take part.

### Data collection

Semi-structured individual interviews were conducted over an interrupted period of 2 weeks (1 week in August 2019 and another in February 2020) to capture the indigenous neonatal feeding and bathing practices of caregivers in the Vhembe District. Participants were interviewed in the outpatient post-natal clinic of the selected hospital for those women who could not accommodate the researcher at their homes because of cultural restrictions. The interviews took place in a private cubicle, with doors closed to prevent any undue intrusions and maintain privacy. For those without such restrictions, the interviews were held at their homes, at scheduled suitable times for them. Indigenous knowledge holders and the THPs were interviewed at their homes using the same interview guide that was used to collect data from mothers and caregivers.

There was no need for either translation or interpretation during the interviews because communication with the participants was in Tshivenda, which is the home language of the participants, the researcher and her assistant. Interviews lasted for 30–40 min per participant and up to an hour for a participant who needed to feed the neonate or change a nappy. The interview guide comprised five open-ended questions for the facilitation of participants’ unconstrained verbal responses (Kumar 2012:111). The interview guide’s questions were not limited to mothers and caregivers because they focused exclusively on indigenous methods of feeding and bathing neonates, which IKHs and the THP are familiar with because of their advisory role on indigenous neonatal feeding and bathing practices.

Probing questions were used to facilitate the prompting and encouragement of interviewees to respond as elaborately as possible. An audio-recorder was used after permission was granted by participants to ensure that the original versions of the interviewees were obtained and retained in an uncontaminated manner (Epstein & Carlin 2012:899). Whilst other participants agreed to be audio recorded, some expressed their discomfort and only allowed the researcher to take notes.

### Data analysis

Data analysis was conducted following the six steps by Creswell (2009) cited in Botma et al. (2010:223). The researcher familiarised herself with the data and subsequently transcribed them verbatim, after which they were then translated from Tshivenda to English with the assistance of the researcher’s academic supervisors as they speak and understand the language perfectly. Names and other associated identifying data were changed to maintain the anonymity of the participants. Themes were generated from the data and were grouped according to their commonalities. The researcher and the supervisors agreed on the themes and sub-themes that had emerged.

### Trustworthiness

The researcher ensured the trustworthiness of the research process by applying the criteria of credibility, dependability, confirmability and transferability. Credibility was ensured by prolonged engagement with the participants in the study field during data collection. Semi-structured individual interviews were conducted for 2 weeks, lasting for a maximum of an hour per participant until data saturation was reached (Birt et al. 2016:1802). Confirmability was ensured with the utilisation of an audio-recorder for capturing participants’ responses in their original and unaltered state. Person and space triangulation was ensured by interviewing participants from different walks of life, that is, women across different age groups and from the different geographic areas within the Makhado Municipality.

### Ethical considerations

The researcher obtained approval to conduct the study from the University of Pretoria, ethics clearance number 107/2019 was issued. Thereafter, approval was received from the Limpopo Provincial Department of Health, the District Executive Officer of Vhembe District, the Chief Executive Officer of the hospital and the facility manager of the clinic. The operational manager at the hospital was notified and given a telephone briefing about the study a week before commencing with participant recruitment and data collection. Permission for interviewing participants at their homes was also received from the local tribal council office. The principle of justice, beneficence and respect for human dignity and the right to privacy and confidentiality were considered.

## Findings

### Demographics

Table 1 summarises the participants’ demographic data in terms of their age, gravidity, parity and educational status. This study revealed that mothers and caregivers use indigenous neonatal feeding and bathing practices across age groups, social standing and level of education. An alphanumeric coding system was used to protect the participants’ identities, consonant with the ethical requirements of safeguarding their privacy, confidentiality and autonomy (Creswell 2017:128). The researcher’s self-designed coding system encapsulated the participants’ number, the research site (whether a clinic or home interview). For instance, P1CM stands for Participant 1 who was interviewed at the clinic and is a mother, P7HM represents Participant 7 who was interviewed at home and is a mother. Furthermore, P17HIKH is Participant 17 whose interview took place at her home, and she is an IKH, whilst P18HTHP stands for Participant 18 interviewed at home and is a THP.

From the interviews, two themes and four sub-themes emerged. Table 2 provides a summary of themes and sub-themes that emerged from the data.

**TABLE 2:** Summary of themes and sub-themes.

Themes	Sub-themes
1. Indigenous neonatal feeding practices	- Feeding of *ntswu* to neonates - Feeding *madi a ngwedi* to neonates - Feeding of *khongodoli* to neonates
2. Indigenous neonatal bathing practices	- Neonatal bathing using *gumululo* or *swandzo*

### Theme 1: Indigenous neonatal feeding practices

It emerged from the study that neonates are offered different types of prelacteal feeds. These feeds include *ntswu* [*Ntswu* is a mixture of boiled water and indigenous herbs *mugwiti* (Combretam molle also known as velvet bushwillow), *mudzidzi* (Artabotrys monteiroae also known as Red Hook-berry) and *musenhze* (Cussonia spicata also known as spiked cabbage (Mabogo 2012:80, 55, 59)], *madi a ngwedi* [water kept in a container with a river stone inside] and *khongodoli* [fine, watery soft porridge made from brown maize meal and some indigenous herbs]. The reasons for offering prelacteal feeds were prevention of illnesses, gut cleansing, promotion of health, prevention of hunger, keeping the neonate calm and promotion of weight gain. The following sub-themes emerged: feeding of *ntswu* to neonates, feeding *madi a ngwedi* to neonates and feeding of *khongodoli* to neonates.

#### Sub-theme 1.1: Feeding of ntswu to neonates

Findings revealed that neonates were fed *ntswu* soon after birth because participants believe that the neonate’s stomach needs cleansing to eliminate the green stools [meconium], as it may cause gastric illnesses. The timing of this feed is as early as 5 days from birth to 6 months. Neonates who had an extended stay at the hospital were given *ntswu* as soon as they arrived home. The reason for giving *ntswu* was expressed as follows:

‘Babies who are breastfed and not given *ntswu* struggle with regurgitation and the green stools does not come out completely and that makes the baby sick.’ (P3CM)

Another participant added:

‘It is important to give *ntswu*, the mother’s breastmilk is not enough and besides, *ntswu* helps with the prevention of *tshilala* [frequent reflux that could develop because of vomiting accompanied by stomach winds and cramps] and diarrhoea. Babies who are breastfed and not given this mixture struggle with regurgitation and the green stools do not come out completely and that my child will cause the baby to be sick.’ (P13HC)

When asked about the dosage and frequency of administering *ntswu,* the following was mentioned:

‘We give it three times a day, in the morning, afternoon and in the evening. You do not have to give too much. You can’t give a whole cup a few tablespoons is enough, and in between the mother should give the breast.’ (P16HIKH)

#### Sub-theme 1.2: Feeding madi a ngwedi to neonates

According to participants, *ngwedi* is a particular stone found in the river. The caregiver or IKH takes the stone from the river, puts it in a container of clean water and keeps it in the water. The neonate should drink this water as soon as it arrives home for 3 days to aid with weight gain and general well-being. A THP who prescribes *madi a ngwedi* to caregivers highlighted that it should not be given for an extended period (more than 3 days) and said:

‘I give madi a *ngwedi* to assist the baby to gain weight, but we give it for only 3 days because we are afraid that the baby will gain too much weight. It also helps to make the neonate’s general health stronger.’ (P18HTHP)

Similarly, participants who support the practice of giving *madi a ngwedi* said:

‘I give *madi a ngwedi* as advised by Vhomaine because I do not want a baby who does not gain weight. Thin babies look like they are sick and people don’t like them. I like fresh babies, they are cute.’ (P8HM)

P8HM was supported by P1CM who continued to feed the neonate in the same manner regardless of the exclusive breastfeeding health information received at the clinic:

‘He [*neonate*] gets thirsty he will lose water because his stools are also loose. Yes the nurses here do tell us that we do not have to give water because breastmilk already contains water, but I do not believe it because I gave all my babies water and they grew up fine.’ (P1CM)

#### Sub-theme 1.3: Feeding khongodoli to neonates

Findings revealed that participants fed *khongodoli* to the neonate whilst awaiting the mother to produce more of the breast milk. The following was observed from the response of the participant:

‘Remember you cannot give the baby the breast milk on the same day that it was born. The milk is not enough and it is not fresh and white it is a bit slippery and yellow it needs to come out so that the baby can get fresh milk. In the meantime, whilst we are waiting for the milk to come we need to make sure that the baby is eating, that is where *khongodoli* comes in, if you don’t feed the baby it will not sleep and will be crying throughout because of hunger. But you will notice that after feeding *khongodoli* baby will calm down and sleep.’ (P18HTHP)

Another participant stated:

‘Neonates are born hungry. Their stomach is empty and full of wind. If the mother does not eat good food during pregnancy, the foetus does not get enough food as well. The neonates should be fed after bathing to prevent crying. When the neonate is fed early, it allows the new mother the time to rest after delivery, as she is exhausted. Giving birth is hard work.’ (P11HC)

In this theme, it was clear that feeding practices were not only for hunger satisfaction but also for protection against illnesses and promotion of health.

### Theme 2: Indigenous neonatal bathing practices

This study further revealed that indigenous neonatal bathing was not only performed for hygienic purposes but also the neonates’ physical strength and protection from evil spirits. The following sub-themes emerged: Neonatal bathing using *gumululo* or *swandzo* (bath water mixed with indigenous herbs) and keeping the neonate warm during and after bathing.

#### Sub-theme 2.1: Bathing the neonatal using *gumululo* or *swandzo*

Apart from bathing the neonate for hygienic purposes, participants mentioned that in their culture, a neonate should receive a special bath for spiritual protection during the first 3 days of life.

One IKH expressed the following when asked what *gumululo*:

‘Gumululo is a special bath that we give to the babies in the first 3–4 days of life. We do not use soap in the early days of life. We mix three traditional medicines with water and boil it, that mixture is then put in the bathwater to bath the baby.’ (P17HIKH)

Another participant intimated as follows in response to why *gumululo* is offered to neonates:

‘This kind of bath helps to make the baby strong, protect the baby from *mirunzi ya vhathu* [evil spirits carried by people with bad intentions], illnesses and it also helps the baby to gain weight. To complete the bath and make it effective for protection we need to make the baby drink a little bit of the water after bathing.’ (P16HIKH)

Similar to feeding practices, bathing is not only for hygienic purposes. It is also implemented for other purposes such as protection from *mirundzi ya vhathu* (evil spirits carried by people), *mutsiko* (general well-being) and relaxation. It is a practice that participants respect and hold with pride.

## Discussion of findings

This study was conducted to explore and describe the indigenous neonatal bathing and feeding practices of mothers and caregivers of Vhembe District Limpopo province. Themes and sub-themes that were identified and are discussed next.

### Indigenous neonatal feeding practices

The indigenous neonatal feeding practices expressed by participants in this study do not only concern the hunger satisfaction of the neonate but also protect the neonate from developing illnesses such as *tshilala* (frequent reflux that is accompanied by stomach winds and cramps), colic as well as gut cleansing. The participants mentioned that *ntswu, mudzidzi, musenhze* and *madi a ngwedi* were fed to the neonate to ensure good health, growth, strength and for gut cleansing, whilst *khongodoli* was given to prevent hunger. Indigenous knowledge holders and the THP concealed the names of other herbs used for indigenous neonatal feeding because certain of their traditional medicines should be kept secret from public knowledge. This could jeopardise the health of new-borns should they have side effects and require hospitalisation; it may be difficult to pinpoint the exact herb that may be causing the ill effects. Participants were urged to consider disclosing all the herbs used in neonatal feeding in the future.

This study revealed that a substantial proportion of participants administered *ntswu* and *khongodoli* to the neonates in honour of older women who serve as caregivers. This is in line with the findings of a systematic review study on the impact of grandparents on breastfeeding rates. According to the study, a grandmother’s negative attitude towards breastfeeding can decrease the incidence of breastfeeding by up to 70%. (Negin et al. 2016:7). This implies that exclusive breastfeeding remains a challenge in rural areas as long as caregivers are excluded from neonatal feeding health education. The timing of *ntswu* is as early as 5 days from birth to 6 months. Other research conducted in South Africa found that early neonatal prelacteal feeding and mixed feeding practices are usual practices in South Africa that are influenced by culture and begin within 1 month of the neonate’s birth (Chaponda, Goon & Hoque 2017:4; Mlambo & Peltzer 2020:7). The practice of giving neonates water and traditional medicines is not unique to the participants of this study, similar findings of giving water and traditional medicines to neonates for gut cleansing were found in another qualitative study in South Africa (Nor et al. 2012:455).

This study revealed that participants felt the need to give *ntswu* to prevent illnesses and ensure the excretion of meconium from the neonate’s gut as informed by their knowledge of indigenous neonatal feeding. Meconium is believed to be a substance that needs to be flushed out of the intestines because it could cause illnesses if it stayed dormant in the gut of a neonate for too long. This study discovered that to this day in Vhembe District, neonatal traditional herbs such as *ntswu* are used to cleanse the neonate’s intestines, to prevent or correct *tshilala* and colic. The practice of using herbs for stomach pain and colic was also found in a Jordanian study that indicated that bottle-feeding boiled herbs or sweetened water for neonates helped them to sleep by reducing gastrointestinal pain and colic (Mrayan et al. 2018:486). The use of herbal drinks to relieve stomach pains was also discovered in Nigeria (Jimoh et al. 2018:40). These are concerning practices as mixed feeding has been proven to cause increased chances of mother to child transmission of human immunodeficiency virus (HIV), acute respiratory and gastrointestinal infections (Kassa 2018:8; Nguyen et al. 2020:126). It may not be safe to use *ntswu* because of the lack of a standardised dosage mechanism; participants were not clear of the exact dose to give. They mentioned giving a tablespoon three times a day regardless of the neonate’s bodyweight; yet this could be harmful to the neonate’s health.

*Khongodoli* is given before the neonate is a week old until solid feeds are introduced. Some participants mentioned that they need to give *khongodoli* as soon as possible because their culture prohibits the feeding of colostrum to the neonate. They believe that colostrum is not fresh milk and that the colour is not as white as milk should be. Therefore, they believe it is not safe for neonate feeding. This finding is similar to the results of some studies conducted in Guatemala and Indonesia, which reported that colostrum was discarded for cultural reasons and was a factor that prevented early initiation of breastfeeding (Patel et al. 2015:11; Sutan & Berkat 2014:8). Colostrum is nutritious and possesses immunological properties that help in the prevention of infections (Motee & Jeewon 2014:62). The practice of discarding colostrum deprives the neonate of its benefits, potentially increasing the neonate’s infection risk. Mugadza (2018:1206) mentioned that in Zimbabwe, thin porridge was not viewed as a form of prelacteal feeding, but as a treatment for gut cleansing to prepare for the breast milk’s arrival. Mlambo and Peltzer (2020:7), in their study, found that feeding soft porridge was the most common practice of neonatal feeding. To some extent, these practices encourage mixed feeding, and it should be discouraged in a culture-sensitive manner and with respect to culture. It could be detrimental to the HIV-infected mothers who must adhere to strict measures of exclusive breastfeeding.

Caregivers, IKHs and THPs all play an advisory role in indigenous neonatal feeding, according to this study. This emphasises the importance of involving them in neonatal feeding communication through health education. As a result, midwives must encourage every mother to bring along a caregiver who will care for her and the neonate following delivery, beginning during the antenatal time and continuing during the post-natal period. Interaction between midwives and caregivers is critical because it allows midwives to have a better understanding of the community’s indigenous infant feeding practices, allowing them to provide culturally sensitive health education.

### Indigenous neonatal bathing practices

Neonatal bathing is amongst the important activities in neonatal care for caregivers. Indigenous neonatal bathing was performed for hygienic and protection purposes. Participants mentioned that they bathe the neonate to be protected from illnesses and evil spirits. *Gumululo* or *swandzo* is an indigenous neonatal bath offered to neonates to remove vernix and make the neonate strong in order to withstand illnesses and attacks from evil spirits. Three herbs are boiled in water and put in the bathwater. Only two herbs were revealed to the researcher which are *musenhze* and *tshisesana* or *gumululo* (Elephantorrhiza elephantine also known as eland’s bean) (Mabogo 2012:127).

*Gumululo* or *swandzo* baths were needed for the protection of the neonate from *mirundzi ya vhathu* and the removal of vernix as it was viewed as taboo. The practice of removing vernix immediately after birth should be discouraged because it deprives the neonate of the anti-infective properties and hydrating effects of vernix on the skin (Visscher & Narendran 2014:145). The practice of indigenous neonatal bathing is not unique to the Vhembe people. Herbal neonatal baths are common in Nigeria, where they are used to protect neonates from illnesses and treatment of other diseases (John et al. 2015:242). In Uganda, the same practice is known, with neonates bathed in a medicated bath called *ekyogero* to bring the neonate luck (Kayom, Kakuru & Kiguli 2015:4; Nankumbi & Muliira 2015:106). From the findings, it was clear that participants believe that neonatal illnesses are not only the results of physiological problems but also a result of external forces such as evil spirits from which neonates should be protected.

In this study, *gumululo* is supposed to be performed immediately after birth to wash off vernix as it is viewed as taboo to keep vernix on the body of the neonate for an extended period after birth. This is impossible because neonates are born in the hospital and the bath is performed as soon as the neonate arrives at home, which is mostly after 6 h, therefore this practice is viewed as harmless. This practice is viewed as complying with the WHO recommendation where bathing should be delayed for 24 h after birth but can be carried out after 6 h for cultural reasons (WHO 2013:25). Participants should be discouraged from making the neonate drink the bathwater as it may contain microbes with the potential of making the neonate sick. The herbs in the bathwater are also not guaranteed to be safe for the neonate’s consumption.

This study revealed that caregivers in Vhembe District do follow the indigenous practices in caring for neonates. It became clear that although not all practices are harmful, some of them could pose health risks to the neonate, such as the practice of mixed feeding and the use of herbs.

### Strengths and limitations of the study

This study revealed that women in Vhembe District follow the indigenous practices in caring for neonates. Although not all practices are harmful, some of them could pose health risks to the neonate, such as the practice of mixed feeding and the use of herbs.

Some possible limiting factors were that the researcher introduced herself as a midwife to participants for transparency purposes. This caused other participants to be reluctant to share information about their neonatal care practices for fear of being criticised. Indigenous knowledge holders and the THP were reluctant to reveal other names of herbs used during the care of the neonate. Only a few were revealed because of the norm of secrecy that is characteristic of many other cultural practices. Consequently, their choice to conceal the names of the herbs was respected.

### Contribution of the study

This study contributes to knowledge creation on indigenous neonatal care practices of mothers and caregivers. The study could make healthcare professionals, particularly midwives, aware of the neonatal feeding and bathing practices carried out by mothers and caregivers in their community. Other studies could be conducted to investigate the hazards associated with the use of herbs for consumption in neonates. This study has the potential to motivate similar studies by other researchers in other settings around South Africa.

### Recommendations

Healthcare professionals, particularly the midwives and the primary healthcare nurses should be informed about indigenous neonatal feeding and bathing practices of the communities they serve to plan relevant culture-sensitive health education for their clients. Information on the benefits and dangers of indigenous neonatal feeding and bathing practices should be included on platforms such as MomConnect. Healthcare professionals should encourage the mothers to bring the caregivers with them to the post-natal visits so that they can also receive health education. Healthcare workers should encourage safe indigenous neonatal care practices to show the community that the biomedical system is willing to work collaboratively with the community and not eliminate the use of indigenous neonatal care practices. This will also encourage the mothers and caregivers to disclose their use of indigenous neonatal care practices.

Information on indigenous knowledge should be incorporated into the midwifery curriculum. Indigenous neonatal care practices should be included as a midwifery module to equip future midwives with relevant information in the provision of culture-sensitive midwifery care. Clinical research ought to be conducted to focus on the usefulness of herbs and other botanical products in illness prevention for neonates. An ethnographic study that will conduct similar research in neighbouring villages outside of the Vhembe District is recommended.

## Conclusions

This study revealed that caregivers use indigenous neonatal feeding and bathing practices across age groups, social standing and level of education. Younger mothers receive guidance from older women in their families or communities. Indigenous neonatal feeding and bathing practices were explored and described. Practices that may compromise the neonates’ well-being were made known to the participants on the spot by the researcher in a culture-sensitive non-judgemental manner.
